# Improving K-12 Teachers’ Acceptance of Open Educational Resources by Open Educational Practices: A Mixed Methods Inquiry

**DOI:** 10.1007/s11423-021-10046-z

**Published:** 2021-09-16

**Authors:** Hengtao Tang, Yu-Ju Lin, Yingxiao Qian

**Affiliations:** 1 Department of Educational Studies, University of South Carolina, 820 Main Street, Columbia, SC 29208 USA; 2 Teaching and Learning Technologies, Purdue University Innovative Learning, West Lafayette, IN 47907 USA; 3 Learning and Cognition Lab, Mary Lou Fulton Teachers College, Arizona State University, Tempe, AZ 85287 USA

**Keywords:** Open educational resources, Open educational practices, K-12 teachers, Renewable assignments, Mixed methods

## Abstract

Teachers in K-12 settings increasingly demand instructional materials beyond textbooks to follow the upward momentum of personalized instruction. Especially during the outbreak of COVID-19, K-12 teachers are forced to quickly adapt to online teaching and thus have more difficulties of delivering personalized instruction in a relatively resource-restraint situation. Open educational resources (OER), allowing teachers to retain, reuse, revise, remix, and redistribute high-quality educational resources at no costs, can be a viable option for teachers. However, the acceptance of OER in K-12 education still remains low. Effective strategies are needed to reinforce teacher intention to adopt OER. This research showcased a two-phase sequential explanatory mixed method inquiry to investigate whether engaging K-12 teachers in open educational practices (OEP)—such as renewable assignments—increased their acceptance of OER. The quantitative phase, referring to the technology acceptance model (TAM), examined the change in factors influencing teachers’ acceptance of OER. The qualitative phase was followed up to provide supplementary perspectives of the quantitative findings. By integrating complementary findings, this research found that OEP increased teachers’ perceived ease of and self-efficacy towards using OER. Although teachers’ intention of implementing OER is not significantly improved, qualitative findings offered additional insights into the benefits of OEP in promoting OER usage and the recommended directions for future effort. Practical implications on improving teachers’ acceptance of OER in K-12 curriculum are discussed at the end.

## Introduction

Globally, there has been a growing desire to move from a “one-size-fits-all” model of K-12 curriculum development to one characterized by differentiated instruction. As a result, K-12 educators are encouraged teach beyond standardized textbooks and personalize the content for their students. This results in teachers’ increasing need for a variety of educational resources beyond textbooks, such as online courseware and supplemental videos, handouts, and assessment rubrics (Blomgren, 2018). However, these resources are usually copyright-restricted. Educators pay for subscription fees to use the resources but are not allowed to reproduce and redistribute them adhering to the copyright. This restriction limits educators’ capacity of delivering differentiated instruction, especially during the recent outbreak of COVID-19. Many K-12 schools are forced to embark on online teaching in such a short time frame in response to the school closure resulted from the pandemic, with very limited time left for teachers to be prepared for this rapid change in course delivery format (Huang et al., 2020). Even school districts are also new to this school-wide online teaching and thus limited resources and professional development support are available for teachers (Van Allen & Katz, 2020). This restraint of resources leads to additional challenges for K-12 teachers in delivering personalized instruction to maintain the effectiveness of online teaching during the pandemic.

Adopting open educational resources (OER) can be an alternative pathway for K-12 teachers to work within this limit. OER are free, open-licensed online educational resources that allow educators to retain, reuse, revise, remix, and redistribute their desired resources (Lin & Tang, 2017). OER are often considered by K-12 educators to be more useful and accessible than traditional textbooks (Blomgren, 2018; Kelly, 2014). With OER, teachers can access more up-to-date materials than those provided in printed textbooks (Kimmons, 2015). In addition, implementing OER in K-12 contexts has been shown to yield positive impacts on students’ outcome and motivation (Wiley et al., 2012). Overall, OER are promising to provide teachers with enriched resources to help each student learn effectively.

However, guiding teachers to adopt OER in K-12 education requires more effort than has hitherto been given (Tang et al., 2020). de los Arcos et al. (2016) raised the concern that most K-12 teachers’ awareness of OER remained deficient to engage with it meaningfully. Barriers such as teachers’ lack of institutional support and their insufficient expertise and willingness in adopting OER also restrained further acceptance of it in K-12 education (Hassall & Lewis, 2016; Kimmons, 2016). To overcome these barriers, open educational practices (OEP) have been integrated in teacher preparation and professional development programs to increase pre-service and in-service teachers’ awareness of OER (Kimmons, 2016; Tang et al., 2020). OEP broadly describe a variety of practices of “creation, use, and reuse of open educational resources (OER) as well as open pedagogies and open sharing of teaching practices” (Cronin, 2017, p. 16). For example, teachers raised their awareness of OER and understood the potential of integrating OER in K-12 classrooms after deliberate practices of selecting, adapting/creating, and then publishing OER (Kimmons, 2016). Bourgonjon et al. (2013) argue that providing teachers with worked-out examples and best practices will help improve their awareness and intentions of technology acceptance. Beyond boosting OER awareness, however, it remains unknown whether, if any, and how OEP can improve teachers’ acceptance of OER in K-12 classrooms.

Therefore, the purpose of this study was to examine whether and how OEP improves K-12 teachers’ acceptance of OER. In particular, this research focused on renewable assignments, an OEP that allow students to adapt OER and then produce and distribute self-generated OER beyond that classroom (Wiley et al., 2017). Examples of renewable assignments can be video tutorials, digital photos (Wiley et al., 2017) and wiki entries (Yaeger & Wolfe, 2018) created by students and published openly for future offerings of the class and beyond. This research was framed by the technology acceptance model (TAM) to understand teacher acceptance of OER (Davis, 1989; Kim et al., 2015). K-12 teachers’ experience with renewable assignments was also discussed. The findings of this research are significant for reinforcing the adoption of OER in K-12 settings.

## Literature Review

### Open Educational Resources

Open Educational Resources (OER) are free educational materials that are openly licensed so that users may manipulate them to suit their personal needs (Tang, 2021a). OER grant users permission to retain, reuse, revise, remix, and redistribute them allowing educators to customize and reproduce a broad collection of materials (Hilton, 2016; Lin & Tang, 2017). In particular, *retain* means users can download and keep a copy of OER. *Reuse* gives users the permission to utilize the whole or a portion of OER. To satisfy users’ personalized needs, OER allow users to make changes to (*revise*) or merge (*remix*) any existing OER (Read et al., 2020). *Redistribute* allows users to share and disseminate OER without any restrictions of copyrights (Hilton, 2020).

Due to their freely available nature, OER has the potential to decrease students’ educational costs without a decline in learning effectiveness. For example, Ross et al. (2018) found that most college students using OpenStax textbooks in a sociology course preferred open textbooks to traditional ones, especially given that using OER was free, accessible, and flexible. Wiley et al. (2012) reported there was no significant difference in standardized test scores between two secondary science courses, one using traditional textbooks and the other OER. Therefore, OER has gained growing popularity as an alternative to decrease student spending on textbook purchases.

### Open Educational Practices and Renewable Assignments

Bourgonjon et al. (2013) argue that providing teachers with worked-out examples and best practices will help improve their awareness and intentions of technology acceptance. Teachers seem more likely to accept technology if they have experience of adapting, creating, and publishing OER. Open educational practices (OEP) afford a contextualized environment for learners to experience the adaptation, production, and publication of OER (Olcott Jr., 2012). The call for OEP has thus risen.

An example of OEPs was to create renewable assignments (Wiley et al., 2017). Renewable assignments engage learners in practices of manipulating OER while completing non-disposable course projects (Jhangiani, 2017; Wiley & Hilton, 2018). For example, students can adapt, create, and publish OER, such as remixing OER for video tutorials and publishing them with Creative Commons licenses (Yaeger & Wolfe, 2018), or participate in open scholarly activities, such as updating Wikipedia entries and annotating articles (Jhangiani, 2017). Preliminary findings from a number of studies indicated that by creating renewable assignments, students boosted course performance (Wiley et al., 2017), enhanced self-efficacy and motivation to learn (Fatayer, 2016; Seraphin et al., 2019), and increased perception of authenticity and interactivity (Yaeger & Wolfe, 2018). Moreover, the merit of renewable assignments goes beyond the class (Wiley et al., 2017). These student-generated OER yield lasting benefits for future students taking the same class and beyond. Any learners interested in the topic can also access and benefit from these student-generated OER. Wiley and Hilton (2018) thus called for incremental pedagogical practices of using renewable assignments and also empirical research on their learning impact.

### K-12 Teachers’ Perception of OER

K-12 teachers who accessed OER highly appreciated the quality and open licenses of OER (Kelly, 2014; Kimmons, 2015; Tang & Bao, 2020). In particular, they showed higher perceived usefulness of OER than college instructors and corporation trainers (Kelly, 2014). Kimmons (2015) attributed this to K-12 teachers having a stronger need to teach out of textbooks. Furthermore, de los Arcos et al. (2016) observed a high tendency of K-12 educators to adapt OER rather than only use OER “as is” (without modification). Kimmons (2015) also found that thirty K-12 educators, as content creators and reviewers, preferred open/adapted textbooks (i.e., open textbooks adapted/customized by teachers) the most, followed by open textbooks (e.g., published in CK-12 and OpenStax) and then traditional textbooks. In other words, teachers appreciate adaptability in the teaching materials that they engage, a fact that makes OER inherently enticing to them.

K-12 teachers also met challenges in using OER (Tang, 2020; Tang & Bao, 2021). First, many K-12 teachers lack the necessary level of awareness about OER to understand how they might use it in their teaching (de los Arcos et al., 2016). Second, K-12 teachers desire updated resources aligned with state standards and students’ grade levels (Kimmons, 2015), but without appropriate maintenance, some OER are outdated or inaccessible (Olcott Jr., 2012). Third, integrating OER required teachers with sufficient self-efficacy and expertise to do so. However, Kelly (2014) found elementary teachers with low self-efficacy in manipulating OER. Hassall and Lewis (2016) added that teachers’ lack of knowledge on how to integrate OER limited adopting OER in their curriculum. These challenges underline the need to understand how to reinforce teachers’ acceptance of OER in K-12 classrooms.

### Teachers’ Acceptance of OER

It is disputed that technology can naturally be adopted in any educational setting without considering teachers’ intentions (Valtonen et al., 2015). Ajzen’s (1991) Theory of Planned Behavior attributes the occurrence of individuals’ behaviors to their behavioral intentions. In K-12 education, teachers’ intention predicts whether they actually integrate technology in their classroom (Anderson et al., 2011). Thus, understanding the factors influencing teachers’ intention for technology acceptance is critical.

Tang et al. (2020) adopted the initial version of the Technology Acceptance Model (TAM) to understand which factors contributed to teachers’ intention of accepting OER. The findings corroborated the arguments in the TAM that teachers’ perceived ease (PE) of using OER and perceived usefulness (PU) of OER determine their intention (IN) of accepting OER, with attitudes (AT) mediating the relationship (Davis, 1989; Tang et al., 2020). Specifically, PE describes teachers’ perception of the effort needed to use OER and it determines teachers’ IN (Tang et al., 2020). PE is also the most fundamental determinant of teachers’ IN since it predicts PU (Kelly, 2014; Kim et al., 2015) and AT (Tang et al., 2020). PU denotes teachers’ perception of how OER can improve their teaching performance (Kim et al., 2015). Research has indicated that whether technology can enhance teaching determines K-12 teachers’ attitude about and intention of technology integration (Anderson et al., 2011; Bourgonjon et al., 2013), such as implementing OER (Tang et al., 2020). In addition, AT describes users’ affective valuation of OER but the valuation depends on users’ PE and PU (Fishbein & Ajzen, 1975). In other words, AT is a mediating variable between teachers’ IN and their PE and PU of using OER (Tang et al., 2020).

Beyond the initial version, TAM was extended to TAM 2 (Venkatesh & Davis, 2000) by adding social influence processes (e.g., subjective norm) and cognitive instrumental processes (e.g., job-fit) as the basis of PU. *Subjective norm* (SN) denotes “a person’s perception that most people who are important to him think he should or should not perform the behavior in question" (Fishbein & Ajzen, 1975, p.302). In other words, people are still likely to exert some unfavorable behaviors if they believe their important referents think they would do so (Ma et al., 2005). Research has indicated that SN determined PU and was also correlated to individuals’ attitudes and behavioral intentions (Valtonen et al., 2015). Another factor relevant to PU is *job-fit* (JF), describing the extent to which users perceive technology is applicable to their jobs (Venkatesh & Davis, 2000). K-12 teachers are more likely to use technology that augments their teaching and student success (Ma et al., 2005). Kim et al. (2015) recognized that job-fit influenced adult learners’ intention of using OER, especially for those with jobs.

Venkatesh and Bala (2008) further proposed the TAM 3 with the addition of self-efficacy (SE) as a determinant for PE. Self-efficacy is rooted in social cognitive theory, describing a person’s “judgment of their capabilities to organize and execute courses of action required to attain designated types of performance” (Bandura, 1986, p.391). Technology-related self-efficacy determined K-12 teachers’ intention of technology acceptance (Anderson et al., 2011). Regarding teachers’ acceptance of OER, teachers’ self-efficacy significantly predicted their PE and then intention of adopting OER (Kelly, 2014).

In all, research has indicated that understanding teachers’ IN is necessary to predict their acceptance of OER in K-12 settings, but it remains unknown whether OEP can help improve teachers’ IN. In addition, evidence regarding the influence of OEP on each factor predicts teachers’ IN is also needed. Therefore, this research focused on teachers’ intention of accepting OER by investigating whether OEP brought in any changes in each predictor. By understanding these predictors, implications on improving teachers’ acceptance of OER can be formulated.

## Research Questions

Teachers’ perception is critical for technology acceptance in K-12 settings. TAM provides a sound framework for understanding teachers’ intention of technology acceptance, but it is noteworthy that teacher perception might be influenced by other factors, such as alignment with standards, grades, and subjects (Kimmons, 2015, 2016). For a comprehensive investigation of teachers’ acceptance of OER, understanding their experience with OER beyond considerations of these TAM dimensions is needed. Therefore, this research focused on two research questions:

1. What is the effect of teachers’ experience with OEP on their acceptance of OER?

2. What is teachers’ experience with implementing OER in K-12 settings?

## Methods

**Fig. 1:**
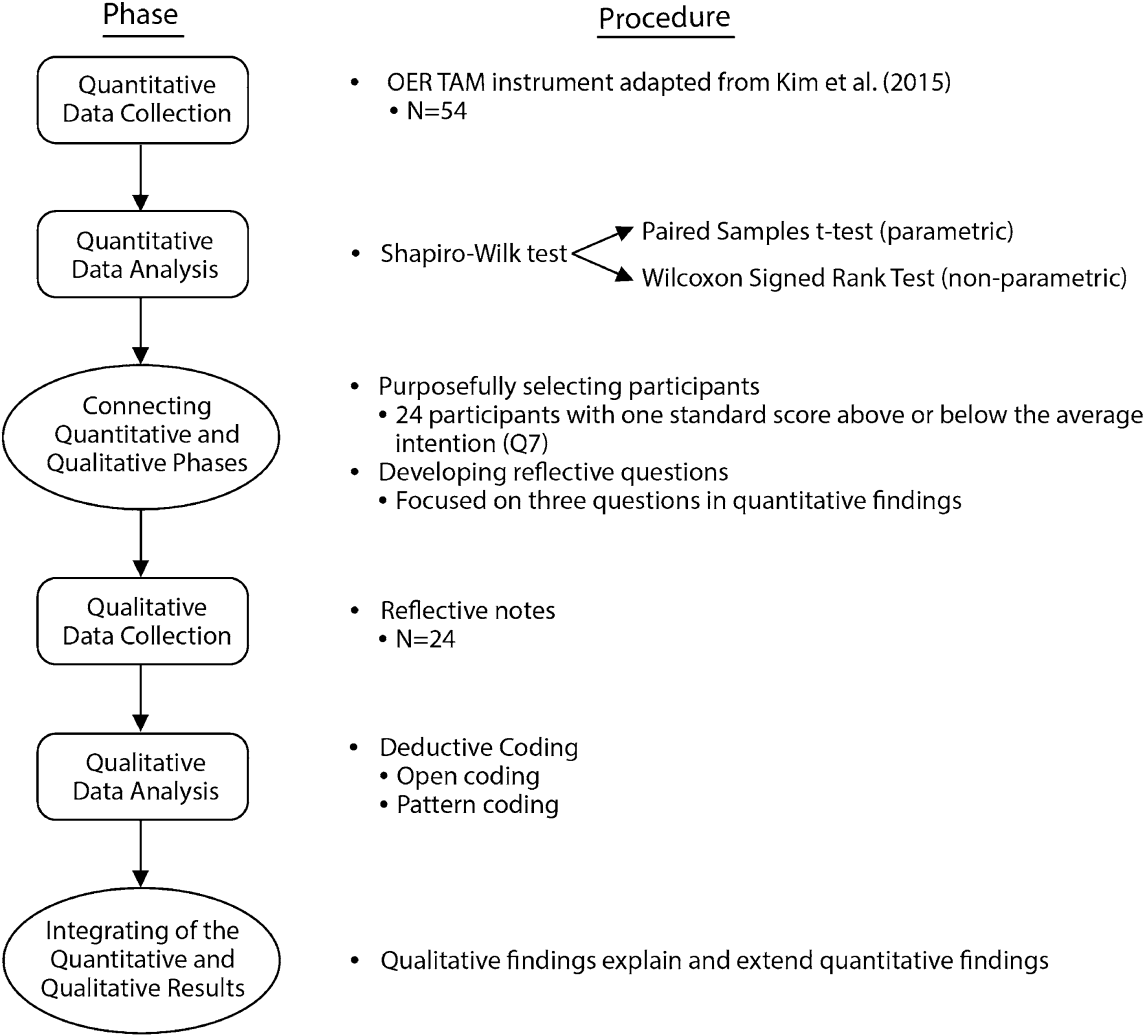
A visual model for the sequential explanatory mixed methods design procedures

A two-phase explanatory sequential mixed method research design (Creswell & Plano Clark, 2017) was applied to comprehensively understand whether and how K-12 teachers’ experience of creating renewable assignments improved their acceptance of OER, by collecting qualitative data to confirm and extend the findings of the quantitative inquiry (see Fig. 1). Specifically, the first phase was the quantitative study, showcasing a one-group pretest–posttest design (Gall et al., 2007). Then, a qualitative inquiry followed to reveal the qualitative story behind quantitative results. To reinforce the rigor of the findings, integration occurred twice when: a) guiding the qualitative data collection anchored to quantitative results, and b) integrating qualitative results to explain and extend quantitative findings (Creswell & Plano Clark, 2017).

### Research Context

This study was conducted in a 16-week online course offered by a graduate program in educational technology at a public university in the southeastern United States. This course had six themed modules with a focus on assessing technology-enhanced learning. Except for the first orientational module and the last reflection module, four content modules focused on understanding, planning, developing, and implementing assessment in technology-enhanced instruction. For this offering, students completed an overarching project by coaching a client to evaluate technology-enhanced learning using assessment materials (e.g., knowledge tests, formative assessments) that integrated OER. Particularly, students who were in-service teachers needed to coach another certified teacher in the same school district. Throughout the course, students cumulatively worked on this overarching project by reviewing effective assessments in K-12 settings, composing assessment plans, developing and implementing OER-based assessments, and then reflecting on the project.

### Participants

A total of three sections were offered for this course, but these sections were merged into one centralized session. Of 78 enrolled students, 68 in-service teachers consented to voluntarily participate in this research, including 57 female teachers. Almost all (*n* = 62) had more than five years of K-12 teaching experience. Five participants had used OER before this course.

### Intervention

The intervention for the one-group pretest–posttest design was to implement renewable assignments as OEP for the participants to gain an authentic experience of searching, adapting/creating, implementing, and publishing OER. The intervention included four phases and participants completed a formative assessment at the end of each phase.

**Fig. 2:**
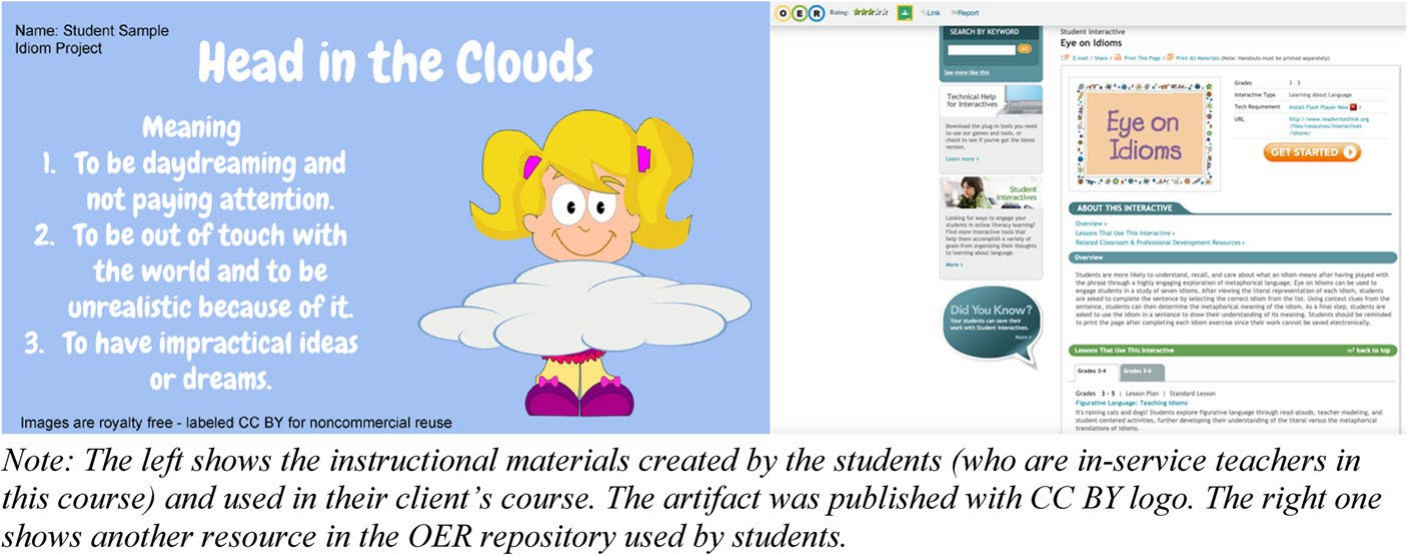
Samples of student work examples

The first phase was to learn about effective assessment. The participants searched existing databases, reviewed samples for effective assessment, and submitted a formative review report for this phase. The second phase was to plan for this OER-based assessment. The participants received instructions on key concepts about OER such as Creative Commons Licenses, 5R principles, and OER repositories (see Appendix 1). Each participant identified their clients during this phase and searched through OER repositories to craft their plans to address the clients’ needs. Then each participant submitted a formative plan about integrating OER aligned with 5R principles to assess technology-enhanced learning. The third phase was to develop and implement the OER-based assessment. The participants reused/revised/remixed existing OER or created new open-licensed assessments tailored to clients’ instructional needs. Then each participant coached their clients to implement the OER-based assessment. Upon the completion of this phase, each participant submitted an implementation report detailing the production and implementation of the OER-based assessments. The last phase was to make revisions and publish the OER-based assessment, paralleling with the reflection module. Each participant received feedback from the instructor and the client to revise the OER-based assessment. Different from disposable assignments, the OER-based assessments developed by each participant (e.g., renewable assignments) were published with open licenses (see Fig. 2) to benefit future students taking this course or educators in a wider community.

### Materials

#### Instrument

The instrument for adult learners’ adoption of OER (Kim et al., 2015, see Appendix 2) was applied to assess the change in factors influencing teachers’ acceptance of OER between pretests and posttests. The seven factors included perceived ease of use (PE), perceived usefulness (PU), attitudes (AT), self-efficacy (SE), subjective norm (SN), job-fit (JF), and intention to use (IN). Each factor was tested on a 5-point Likert scale with responses recorded as (1) “strongly disagree”, (2) “disagree”, (3) “neutral”, (4) “agree”, and (5) “strongly agree”. Specifically, PE was assessed by three items with specifications on the effort level needed for teachers to find, access, and use OER. PU was tested by three items on the extent to which teachers’ productivity and performance could be improved with the use of OER. Three items within AT were used to determine whether teachers were positive or negative about using OER. SE was measured by two items addressing teachers’ readiness and confidence of using OER. SN consisted of three items to measure teachers’ perception and belief about the use of OER. JF was measured by four items considering the degree to which the use of OER was applicable to their teaching practices. Three items relevant to IN evaluated teachers’ intention of using OER. The reliability of the instrument was confirmed as Cronbach's alpha value (*α* = 0.95) was acceptable.

#### Reflection Notes

During the last week, students reflected on the assessment project by responding to reflective questions. Those questions were developed to explain and extend the quantitative findings, with a focus on significant results and results inconsistent to the literature (Creswell & Plano Clark, 2017).

### Data Collection and Analysis

This study included two phases of inquiries in a sequential timing wherein the quantitative phase occurred before qualitative inquiry. Before the data collection, Institutional Review Board approval was obtained.

#### Quantitative Phase

The instruments were sent via a Qualtrics link in Module 1 (pre-tests) and Module 5 (post-tests). Participants voluntarily completed the instrument and had the right to withdraw at any time. In the end, 54 participants completed both pretests and posttests, and only their responses were recorded for analysis.

A Shapiro–Wilk test was administered to determine whether the assumptions for a paired sample t-test were satisfied for each factor. Then the paired sample t-test was conducted for those factors with normal distributions to compare the difference between the pretest and posttest responses. For the other factors without normal distributions, a non-parametric Wilcoxon signed-rank test was conducted. Cohen’s *d* (parametric) or the rank-biserial correlation *r* (non-parametric) was calculated to understand the magnitude of the effect size for each change, if any.

#### Qualitative Phase

Each student taking the course completed reflection notes in Module 6, but determining participants included in the qualitative phase was contingent upon the quantitative results. Participants whose intentions of accepting OER extremely increased or decreased (i.e., *z* score on responses to IU items) were selected (Creswell & Plano Clark, 2017). Reflective notes were used as a measure to collect qualitative responses from the selected participants.

A top-down deductive coding process (Fereday & Muir‐Cochrane, 2006) was employed to seek codes and patterns from the selected participants’ reflective notes. Codes are labels assigned to different chunks of data to represent their meaning as perceived by researchers (Roulston, 2010). Specifically, a priori template of codes was identified before the coding procedure initiated based on the TAM theories (Davis, 1989; Venkatesh & Bala, 2008; Venkatesh & Davis, 2000) and OER works (Hilton, 2016, 2020). Sample codes included “attitudes”, “intention”, and “self-efficacy”, and “remix”, “revise” and “open licenses”. The coding procedure included two cycles: (1) open coding to generate codes for each sentence; and (2) pattern coding to aggregate relevant codes into categories and themes (Saldaña, 2015). For open coding, two researchers with OER expertise individually read each document and assigned codes to each segment in the data. Inter-coder reliability was measured by a Cohen’s Kappa (*k* = 0.878) and the reliability was acceptable (Cohen, 1960). Then the two researchers discussed all the disparities in the codes and made edits to reach a 100% agreement on all the 352 coded segments. The second cycle was pattern coding, wherein the two researchers collaboratively aggregated relevant codes into patterns to elicit themes (Saldaña, 2015). A theme “at minimum describes and organizes the possible observations and at maximum interprets aspects of the phenomenon” (Boyatzis, 1998, p. 161). To ensure rigor and the trustworthiness, constant comparison (Patton, 2002) was applied to assure categories and themes were grounded in the data. Then member checking (Merriam, 1998) was performed with five of the participants and their responses confirmed that the findings reflected their perceptions. The qualitative findings are presented in themes followed by associated categories.

## Results

### What is the Effect of K-12 Teachers’ Experience with OEP on Their Acceptance of OER?

Shapiro–Wilk test results indicated that PU (p = .136) and SN (p = .11) satisfied the assumptions of normality, but the other five factors did not (p < .05). For PU and SN, a paired samples t-test was conducted to determine whether each of the two factors significantly changed after teachers’ experience with OEP. The results (see Table 1) suggested no significant changes on teachers’ PU [*t*(53) = 0.25, *p* = .81], and SN [*t*(53) = 1.61, *p* = .11]. For the other five factors, a Wilcoxon signed-rank test was conducted. The results indicated that teachers’ PE and SE towards OER significantly improved after the OEP experience. According to Cohen (1992), the effect size is small if *r* is below 0.1. The effect sizes represent medium and large if their *r* values are respectively above 0.3 and 0.5. Both effect sizes in our study range between small and medium. Specifically, post-test scores for PE (Mdn = 12.00) were significantly higher than that of pre-tests (Mdn = 11.00), *Z* = 2.68, *p* = .01, *r* = 0.26. For SE, post-test scores (Mdn = 8.00) also significantly improved from pre-tests (Mdn = 8.00), *Z* = 2.54, *p* = .01, *r* = 0.25. Additionally, though participants’ IN was not significantly improved, the median of this factor increased after the OEP experience.

**Table 1:** Quantitative results of factors influencing teachers’ acceptance of OER

Factors	Results of paired samples t-test							
	Pre-tests (*N* = 54)		Post-tests (*N* = 54)					
	M	SD	M	SD	*t*	df	*p*	Cohen’s *d*
PU	11.80	2.21	11.89	2.69	0.25	53	0.81	0.03
SN	10.28	2.10	10.72	2.12	1.61	53	0.11	0.22

	Results of Wilcoxon signed-rank test							
	Mdn	SD	Mdn	SD	*Z*	df	*p*	*r*
PE	11.00	2.35	12.00	2.47	2.68	53	0.01*	0.26
AT	12.00	2.48	12.00	2.63	0.44	53	0.66	0.04
JF	15.00	3.37	16.00	3.26	0.61	53	0.54	0.06
SE	8.00	2.24	8.00	1.84	2.54	53	0.01*	0.25
IN	10.00	2.36	11.00	2.88	0.00	53	1.00	0.00

*p < 0.05 indicates the result is significant

### Integration I: Anchoring the Qualitative Data Collection to Quantitative Results

The quantitative results revealed that three questions needed additional qualitative evidence, including (1) How did the OEP experience improve teachers’ acceptance of OER? (2) What were teachers’ perceived challenges in accepting OER? and (3) What were teachers’ challenges with OEP? These three questions were thus the sensitizing concepts that guided qualitative data collection (e.g., developing reflective questions, selecting participants) and directed the deductive coding procedures (Bowen, 2006).

#### Developing Reflective Questions

To answer these three questions, reflective questions were developed with a focus on understanding participants’ experience with OEP, perception of OER, and desired support to implement OER in K-12 settings. Only reflection notes submitted by those participants with an extreme change in intentions of accepting OER were included.

#### Participant Selection

To select participants with extreme changes, *z* scores on the change in IN items were calculated for each participant (Creswell & Plano Clark, 2017). A total of 24 participants, whose responses were more than one deviation above (*n* = 14) or below (*n* = 10) the average, were included in the qualitative phase (see Appendix 3 for participants’ demographics and survey response records). Their reflection notes were retained and analyzed for qualitative data analysis.

### What is Teachers’ Experience with Implementing OER in K-12 Settings?

Three (sub-)questions developed from the quantitative results were investigated to answer this research question. The findings on each sub-question are reported accordingly.

#### How Did the OEP Experience Improve Teachers’ Acceptance of OER?

Three themes emerged in describing how teachers’ OEP experience altered their perception of implementing OER: (1) developed an authentic awareness of OER; (2) increased self-efficacy about using OER; (3) cultivated a positive attitude towards OER.

##### Developed an Authentic Awareness of OER

Most participants (*n* = 15) indicated that this OEP experience provided a contextualized opportunity of customizing OER and further developed an authentic awareness of OER. This had been the first time that most participants used OER, and this contextualized experience allowed them to understand OER, especially the benefits of OER in an authentic manner. Even for those who had used OER, this OEP experience reinforced their awareness of OER via directly searching and adapting OER for the client.

[Previously] I did not invest much time into researching the use of OERs. After taking this class, however, I am now understanding the benefit of using OERs. (P7)

OER allow for a more tailored experience with the facilitation of information and would in turn allow teachers and students to work together in more authentic learning experiences. (P12)

While I had had some experience implementing them in the classroom, I had no formal introduction to them or their background or purpose. It was not until enrolling in this course that I learned that they were more than some found resource to help the math teachers in my building with instruction/assessment planning. (P23)

##### Increased Self-efficacy About Using OER

More than half of the participants (*n* = *14*) perceived that OEP increased their competence to access and use OER. Participants enhanced their confidence in teaching with OER and also assisting other teachers in using OER.

After completing this course, I feel much more confident with using OERs as a learner and as a teacher. (P11)

I thoroughly enjoyed working with another teacher to help her implement a technology-based assessment using OERs. This class and the assignments we completed gave me the confidence I need to offer my assistance to other teachers. (P14)

After this course has ended, I hope to use my newly gained skills to explore more OERs and help other teachers in my school implement them in their classrooms. (P22)

##### Cultivated a Positive Attitude Towards OER

Most participants (*n* = 16) cultivated a positive attitude towards using OER via participating in the OEP. Even two participants whose intention of accepting OER decreased were also positive about using OER and planned to speak with the leadership team to request support for OER implementation. Some developed positive attitudes about OER since they enjoyed implementing OER. Others were positive about OER because of the lasting benefit of renewable assignments. They planned to share their work to a broader community in order to benefit future students, colleagues, and other educators.

After taking this course, it has also inspired me to create an online OER for my students, future students, or even other educators who would like access to the materials and resources that I may have. (P5)

Lastly, I discovered the inner desire to want to create my own OER for my content, so as to encourage and inspire others and also to allow for my students to access all of the information about my course at their own time and place. (P6)

Using OERs in my project was a great experience. I enjoyed finding all different resources that I can use freely in my classroom. (P11)

My goal is to speak with the administration team to discuss professional development for our staff on OER Commons and implementing assessments that will engage and motivate all of our students. (P17)

#### What were Teachers’ Perceived Challenges in Accepting OER?

Four themes emerged in regard to teachers’ challenges in accepting OER, including: (1) time needed to sift through applicable OER; (2) the inconsistent quality of OER; (3) school districts’ discouragement of OER; and (4) lack of positive attitudes.

##### Time Needed to Sift Through Applicable OER

Participants (*n* = 13) found it time-consuming to find and adapt OER tailored to their needs, which decreased their PE towards OER. This challenge was mainly attributed to participants’ struggles in narrowing down their search. Despite the PE in finding and accessing various OER, participants struggled selecting an applicable resource from all that were available. Participants also found it time-consuming to determine whether a resource was OER due to insufficient knowledge about open licensing.

Although using OERs to gather information was easy, I did find the task of implementing OERs into the development of the assessment task to be extremely challenging. This was something I struggled with and did not do efficiently. I believe that the biggest issue I had with locating these OERs to incorporate into my assessment task was my lack of understanding on how to find valid OERs. (P15)

However, searching online for resources can be time consuming, especially … finding OERs that fit within my learning objectives was not an easy task. Another issue with OERs is figuring out the permissions for a specific resource: what am I allowed to do with a resource once I’ve found it, and how do I know it’s an OER? (P17)

##### The Inconsistent Quality of OER

Most participants (*n* = 17) were concerned about the unstable quality of OER, which decreased teachers’ PU of OER. Participants’ perception of whether OER fit their teaching decreased because some OER were unaligned with curriculum standards or inappropriate for students’ grade or age. This caused teachers to spend significant time adapting OER, “nullifying the time-saving benefit of using an OER” (P5). Participants also revealed some OER were not readily maintained or updated as they found many outdated, broken, or missing links. Another concern about the quality of OER was some OER were not validated because anyone could publish OER without any quality assurance.

It really depends on the particular OER as to the quality—there is a lot of variety, and not everything fits with your curriculum or learners. Some things require a lot of modification, nullifying the time-saving benefit of using an OER. (P5)

While searching for OER, I did discover many resources that were no longer available. Many links were no longer accessible. It seems as if the resources come and go, and the OER repositories are not updated when resources are no longer available (P13).

While I do see the quality to be a pro, it should also be noted that at times the quality can be unreliable, because many sources can input data (P19).

One concern that was raised in our online session is the validity of the resources available. There seems to be some concern in the educational community about the use of OERs because there is fear that they might not be quality resources and could contain false information or even viruses that can infect computers (P24).

##### School Districts’ Discouragement of OER

Participants (*n* = 12) discussed a lack of support from the district in using OER, which decreased their perceived SN and PU about OER. Some participants were required to use educational resources provided or approved by the school district, and those “vetted” resources were perceived more useful than OER in addressing teachers’ needs aligned with standards. Some OER excluded from district-approved resources were blocked, which further decreased teachers’ PU of OER.

Most school districts and counties are attempting to provide their teachers with the appropriate resources necessary to complete their instructional goals. In addition, there is a wealth of websites and applications that are much more accessible and easy to navigate. I just can't imagine a public or private teacher navigating the OER unless it is absolutely necessary. (P2)

My school administrators are requiring that we use "vetted" resources. Only resources approved by the GADOE or my school are encouraged at this time, so I am not really able to use much of what is available through OERs at this time. (P20)

One drawback for me is that, in an effort to protect students, the technology department has blocked many of the open resources or parts of them that could be beneficial. (P23)

##### Lack of Positive Attitude

Participants (*n* = 8) showed a lack of positive attitudes about OER. Some participants had negative attitudes about OER due to their struggle in searching for or sifting through appropriate OER. Other participants were also not positive about using OER due to low appraisals of OERs’ usefulness compared to existing resources accessible to them.

I believe I could have done better at keeping an open mind about OERs. Because of the difficulty I experienced in locating resources, I quickly dismissed OERs as of no value when it comes to disabilities. (P17)

[OER] worked perfectly but as an educator, I already have a tremendous amount of resources at my disposal that are free and accessible therefore I will not be returning to OERs. (P22)

I must say, OERs were not something that I actively thought much about. To be honest, they were useful to me when I needed them, but I never put much thought in how they appeared online. I certainly didn’t think about adding to them! (P24)

#### What were Teachers’ Challenges with OEP?

Two themes emerged regarding teachers’ challenges with their OEP experience, including: (1) time constraints; and (2) a lack of prior experience.

##### Time Constraints

Most participants (*n* = 19) expressed the concern about time constraints in completing all the required activities in OEP. Participants indicated that understanding and developing proficiency in searching and adapting OER was time-consuming, and the length of the course duration was not sufficient to accomplish it.

I felt that there were less OERs that focused on AP Physics and Physics content. I wish I had more time to explore them. (P4)

Personally, I feel that I need more time to research OER for my subjects in order to benefit from their use. (P18).

##### A Lack of Prior Experience

Another challenge described by most participants (*n* = 15) in completing renewable assignments was the lack of prior experience with OER. Participants without awareness of, or experience with, OER struggled in finding and adapting appropriate OER tailored to their needs. They demanded more in-depth research to efficiently understand and use OER and then complete the required activities.

I believe this was the most challenging assignment for me because I had to work and adapt with not only a colleague, but an entire class using something (OER) I had never previously used before. (P19)

Coaching the client throughout this lesson was not too challenging since I have experience teaching. However, integrating Open Educational Resources (OER) was new to me and required some in-depth research (P22).

### Integration II: Integrating Qualitative and Quantitative Results

**Fig. 3:**
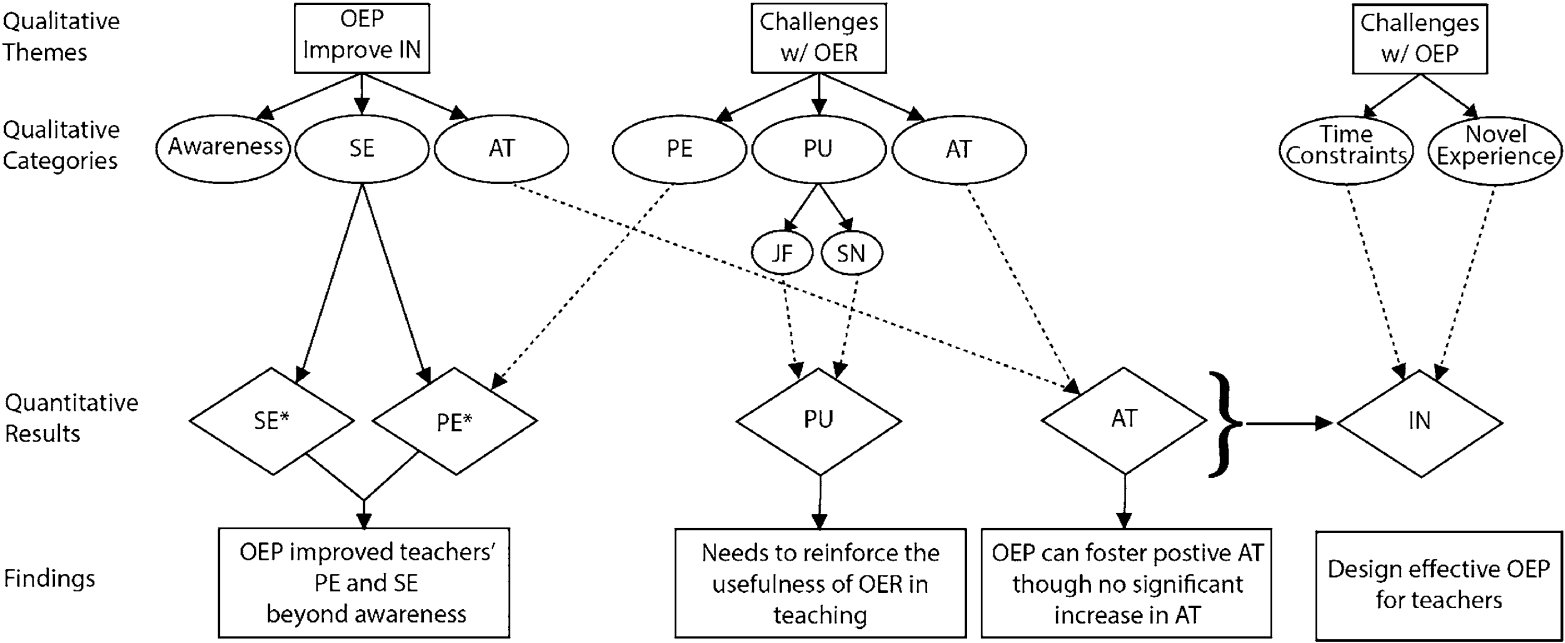
A visual representation of findings by integrating quantitative and qualitative analysis. *indicates significant increase in SE & PE, otherwise other factors (e.g., PU, AT, & IN) have no significant change. *PE* Perceived ease of using OER, *SE* self-efficacy about using OER, *PU* perceived usefulness of using OER, *JF* job-fit of using OER for teaching, *SN* subjective norms about using OER, *AT* attitude about using OER, *IN* intention of using OER. Solid lines between qualitative categories and quantitative results indicate positive influence, but dash lines indicate challenges

The second integration was conducted to explain and extend quantitative findings with qualitative results (see Fig. 3). Specifically, categories in qualitative findings were compared and contrasted with quantitative results. For example, quantitative results indicated participants’ PE and SE of using OER increased after the OEP experience. The category, *increased self-efficacy about using OER,* explained this occurred because participants gained an authentic experience of searching, adapting, and publishing OER. This authentic experience helped them become aware of OER, improve self-efficacy of finding and using OER, and reinforce PE of using OER. However, the increase in participants’ PE of OER was limited to a small range. The category, *time needed to sift through applicable OER*, explained that OEP’s influence on teachers’ PE were rescinded by the extensive time needed to search for appropriate OER.

Qualitative results also provided supplementary insights on why OEP did not improve participants’ PU, AT, and IN. Specifically, for PU, the inconsistent quality of OER decreased participants’ perceived JF towards OER and the school district’s deprecation of OER reduced participants’ SN about OER. For AT, participants’ struggles yielded negative attitudes towards implementing OER although OEP helped cultivate positive attitudes. Participants also had to undergo time constraints and inexperience with OEP. Overall, the integrated results explained why participants’ IN of accepting OER did not significantly increase after the OEP experience.

## Discussion

This study showcased a two-phase explanatory sequential mixed methods study to understand how K-12 teachers’ experience with OEP, specifically renewable assignments, influenced their acceptance of OER, drawing inference from the TAM (Davis, 1989; Tang et al., 2020). The quantitative phase examined whether teachers’ acceptance of OER changed after creating renewable assignments. To explain and extend these quantitative findings, the qualitative phase followed to inquire how OEP influenced teachers’ acceptance of OER and their associated struggles. By integrating complementary findings, this research contributed to implementing OER in K-12 education.

Although teachers’ intentions to use OER did not significantly increase, the changes in teachers’ perception driven by the experience of creating renewable assignments cannot be overshadowed. For example, the research found teachers’ PE and SE about using OER improved, extending the benefits of OEP beyond increasing teachers’ awareness of OER (Kimmons, 2016). Echoing Bourgonjon et al. (2013), qualitative results added that teachers’ authentic experience of searching, adapting, and sharing OER afforded by the OEP might help improve teachers’ PE and SE. This authentic experience provided teachers with worked-out examples and calibrated their self-efficacy towards integrating OER in teaching. In addition, this experience prepared teachers to implement OER in teaching practices, addressing the barrier of teachers’ insufficient expertise (Hassall & Lewis, 2016). We thus speculate that affording a contextualized opportunity for teachers to gain awareness of OER and then implement OER in an authentic context might be an initial step to improve teachers’ self-efficacy and readiness of implementing OER (Olcott Jr., 2012; Wiley & Hilton, 2018; Wiley et al., 2017; Yaeger & Wolfe, 2018). The result might also not conceal the likelihood of OEP (e.g., renewable assignments) in cultivating everlasting benefits beyond the class (Wiley & Hilton, 2018; Wiley et al., 2017). Especially, teachers who had a positive OEP experience in this study decided to extend the benefits of OER by advocating for the use of OER in their school district and sharing OER with a broader community.

It is surprising that teachers’ PU of OER did not significantly improve, even with a decrease in the mean score, after the OEP experience. Qualitative results provided supplemental insights accounting for this finding with a focus on the limitation of OER. First, some teachers who perceived OER as useful also chose not to implement OER because they were satisfied with resources currently accessible online or vetted by the school district. Without support from the school district, convincing teachers to implement OER rather than those resources vetted by the school district is difficult. Second, participants’ responses echoed the concerns in previous studies (Hassall & Lewis, 2016; Olcott Jr., 2012) that many OER were not readily maintained or updated to address the current needs in K-12 classrooms. Kimmons (2015) argued that K-12 considered OER as a viable alternative for updated materials beyond traditional textbooks, but without appropriate maintenance, teachers’ PU of OER tended to decrease. This renders the need to establish a mechanism that maintains the sustainability of existing OER, such as open scholarly activities (Jhangiani, 2017) similar to Wikipedia that gives users access to edit and update published OER in the repository. Unfortunately, this proposed mechanism might raise another concern on whether or not OER could be validated if everyone can publish and edit them. Teachers expressed their concerns about false sources of information or insecure resources being detrimental to K-12 students. Teachers also found it challenging and time-consuming to search for resources well aligned with state standards or appropriate for their students. An efficient quality assurance system in lieu of the textbook publishing system is urgently needed for OER, reiterating the proposition by Kimmons (2015, 2016).

In addition, teachers’ attitudes towards accepting OER did not significantly change by participating in OEP. Our understanding about the reasons why the attitudinal change was not engendered was grounded in the TAM (Davis, 1989; Venkatesh & Davis, 2000). We speculate that teachers’ attitudinal change was also influenced by their PE and PU. Due to teachers’ perceived challenges of implementing OER (e.g., the unstable quality of OER, significant time needed to sift through applicable OER), teachers’ positive attitude fostered through OEP might be undermined.

### Practical Implications

This mixed method study provided practical implications on implementing OEP to improve K-12 teachers’ acceptance of OER, especially in response to the rapid increase in online teaching during the outbreak of COVID-19.

First, teacher educators might consider implementing OEP in teacher education or professional development programs to foster preliminary readiness and intention of using OER (Kelly, 2014; Tang et al., 2020; Wiley et al., 2017). The upsurge of online teaching during the outbreak of COVID-19 created an opportunity for further growth in the adoption of OER in K-12 settings (Van Allen & Katz, 2020). Most K-12 school districts have never been exposed to this type of school-wide online instruction and have limited experience in overseeing the rapid transition into online teaching. Accordingly, school districts have limited capacity of providing vetted resources to address the need for personalized instruction during the pandemic. Teachers are still in great need of digital resources, especially open-licensed resources, during the shift to online teaching (Huang et al., 2020), so teachers are encouraged to create, adapt, and publish OER in order to help each student learn effectively. The findings of this research revealed that OEP as worked examples allowed teachers to gain an authentic experience with OER and improved teachers’ self-efficacy and PE of using OER. It is also noteworthy that an elaborated design is needed to assure effective OEP experiences. Specifically, including formative assessment as a milestone for each phase of the OEP experience may scaffold teachers to reflect on worked examples and receive formative feedback. In addition, time constraints and teachers’ lack of prior experience with OER hampered teachers’ experience with OEP, so school leaders and professional development specialists might allocate teachers with sufficient time to work on OEP activities.

Second, the effort to adopt OER in K-12 education requires school districts’ involvement. Teachers’ perception of OER was influenced by the administrator’s attitude and the policy enacted in the school district. It may help further promote OER adoption if K-12 school districts participate in open education initiatives. Exemplary initiatives such as #GoOpen, launched by the United States Department of Education (n.d.) to advocate for the implementation of open textbooks in partnership school districts, might involve K-12 school districts in increasing teachers’ implementation of OER.

Third, educational authority may fuel cross-sector effort to establish a quality assurance system for OER. The inconsistent quality of OER hinders teachers’ appraisal of its usefulness for teaching and thwarts teachers’ intention of using OER. Ensuring the quality, validity, and sustainability of OER is thus necessary. To make OER grade-appropriate and subject-appropriate, OER creators might align production with Common Core standards and explicitly tag resources when redistributing or publishing them.

Methodologically, this mixed method inquiry presented implications for future scholarly inquires on OER adoption in K-12 settings. Future inquiries are recommended to go beyond the investigations on the effectiveness of OER on learning and teaching (Tang, 2021b), seeking for complementary quantitative and qualitative insights to explore efficient methods of OER implementation in K-12 settings (Creswell & Plano Clark, 2017).

### Limitations and Future Research

This research was restrained by several limitations. For example, renewable assignments, as relatively new course activities for most K-12 educators, required instructors’ facilitation to ensure the progress aligned with research design (Jhangiani, 2017). The frequency of participants’ interaction with instructors might alter some results in this research. Furthermore, subjective bias might be involved in the interpretation of qualitative findings based on deductive analysis (Fereday & Muir‐Cochrane, 2006). The small sample size (*N* = 68) and the effect sizes ranging between small and medium may lead to our findings being less generalized, although the studies investigating OER adoption in the K-12 context is scarce. In addition, the instrument used in this research was adopted from a study investigating Korean adult learners’ perceptions of accepting OER (Kim et al., 2015). Though the validity of the instrument was confirmed, cultural and contextual differences might bring in additional threats to the rigors of this research. For future research, it is highly recommended to further validate the instrument used to assess K-12 educators’ acceptance of OER referring to TAM. A future study with a large sample size and a validated instrument may help improve the effect size to some extent. Additionally, future study may consider integrating data sources such as interviews and surveys with open-ended questions that are no involved the course instructors to further improve the rigor of this study. Also, for future research on OEPs, researchers must make sure they are assigning enough time for students to familiarize themselves with OER and complete the required projects.

## Appendix 1

Screen captures of the samples for OER instruction and assignment guidelines (CSS style was removed to withhold the institutional information).

1. A sample of the assignment guidelines.

(2) A sample of the instruction on OER.

(3) A sample of the instruction on OER repositories for K-12 educators.

## Appendix 2

The instrument for adult learners’ adoption of OER adapted from Kim et al. (2015)

**Table:**

Factors	Items
Perceived ease of use OERs	1.1 I find OERs on the internet easy to use
	1.2 It is easy to become skillful at using OERs
	1.3 It is easy to access information in OERs
Perceived usefulness of OERs	2.1 Use of OER will improve academic productivity
	2.2 Use of OER will increase my learning performance
	2.3 Use of OER enabled me to accomplish tasks more quickly
Attitude towards OERs	3.1 I am positive towards OERs
	3.2 Studying through OER is a good idea
	3.3 Studying through OER is a wise idea
Subjective norm regarding OERs	4.1 I like using OER based on the similarity between my values and society's values regarding its use
	4.2 What OERs stands for is important for me as a learner
	4.3 I think that my peers often use OERs
Job-fit of OERs	5.1 OER will have an effect on my job performance
	5.2 OER can decrease the time needed for important job responsibilities
	5.3 OER can increase the effectiveness of performing job tasks
	5.4 OER can increase the quantity of output for the same amount of effort
Self-efficacy about OERs	6.1 I have the necessary skills for using OERs
	6.2 I feel confident in finding information in OERs
Intention to Use OERs	7.1 I intend to check news about OERs frequently
	7.2 I will always try to use OERs in my daily life
	7.3 I intend to continue using OERs in the future

## Appendix 3

The list of the participants included in the qualitative phase based on the quantitative results.

**Table:**

	Demographics				Teachers’ intention to use OER			
	Gender	Subject	Grade	OER use	Pre-test (M = 10.59)	Post-test (M = 10.46)	Comparisons	*z* Score of comparison
P1	M	Math	K9-10	N	10	14	4	1.48
P2	F	Social studies	K9-12	N	9	13	4	1.48
P3	F	Library media	PreK-K6	N	9	13	4	1.48
P4	F	All subjects	K2	N	9	13	4	1.48
P5	F	All subjects	K1	N	9	12	3	1.12
P6	F	Science	K6	N	9	12	3	1.12
P7	F	Social studies	K7	Y	3	6	3	1.12
P8	M	Science	K6-12	N	11	14	3	1.12
P9	M	Social studies	K8	N	12	15	3	1.12
P10	F	All subjects	K5	N	9	12	3	1.12
P11	F	Math	K6-8	Y	12	15	3	1.12
P12	F	Library media	K-5	N	9	12	3	1.12
P13	F	Reading	K6	N	9	12	3	1.12
P14	F	Math & science	K6	N	12	15	3	1.12
P15	F	Science	K10-12	N	15	12	− 3	− 1.03
P16	F	Library media	K5	N	12	9	− 3	− 1.03
P17	F	Special education	K6-8	N	15	12	− 3	− 1.03
P18	F	Math	K7	N	9	6	− 3	− 1.03
P19	F	Science	K8	N	10	7	− 3	− 1.03
P20	F	Math	K1-4	N	10	6	− 4	− 1.39
P21	F	Math	K7	Y	12	8	− 4	− 1.39
P22	F	Music	K4-12	N	14	10	− 4	− 1.39
P23	F	Math	K9-12	N	11	6	− 5	− 1.75
P24	F	Library media	K5	N	12	3	− 9	− 3.18
